# Research on optimization algorithms for localization and capacity determination of chargers considering the spatiotemporal distribution of electric vehicles

**DOI:** 10.1038/s41598-024-66231-6

**Published:** 2024-07-02

**Authors:** Mingzhen Li, Zeyang Tang, Wei Rao, Yiwen Li, Xinsong Zhang, Cheng Wang

**Affiliations:** 1 School of Electrical Engineering and Automation, Nantong University, Nantong, 226019 China; 2 State Grid Hubei Electric Power Research Institute, Wuhan, 430077 China; 3 School of Life Sciences, Nantong University, Nantong, 226019 China

**Keywords:** Power distribution, Electrical and electronic engineering

## Abstract

The optimized layout of electric vehicle (EV) chargers is not only crucial for users' convenience but also a key element in urban sustainable development, energy transition, and the promotion of new energy vehicles. In order to provide a basis for the problem of localization and capacity determination of chargers and compare the merits of several mainstream algorithms, this paper first establishes an optimization model with the objective of minimizing the total investment cost of all the chargers and the constraint of meeting the charging demands of all electric vehicles. Optimizations were performed using genetic algorithm (GA), surrogate optimization algorithm (SOA), and mixed integer linear programming (MILP) algorithm, respectively. In the case of using MILP, the original nonlinear optimization problem was transformed into a linear problem. In the planning of city-level EV chargers, MILP took 14182.57 s to calculate the minimum cost of 34.62 million yuan. After retaining only 10% of the original data amount, SOA took 87651.34 s to calculate the minimum cost of 3.01 million yuan. The results indicate that GA is prone to falling into local optima and is not suitable for large-scale optimization problems. SOA, on the other hand, requires significant memory consumption, so the issue of memory usage needs to be carefully considered when using it directly. Although MILP is only applicable to linear programming problems, it has the advantages of lower memory usage and higher reliability if the problem can be transformed into a linear one.

## Introduction

### Background and motivation

In recent years, with the technological development in the field of electric vehicle (EV) and the improvement of people’s living, the number of EVs has increased rapidly. The large-scale access of EVs will have an important impact on power grid operation, transportation planning and other aspects. By the end of 2023, the total number of new energy vehicles in China reached 20.41 million, accounting for 6.07% of the total number of vehicles. Among them, the total number of pure electric vehicles reached 15.52 million, accounting for 76.04% of the total number of new energy vehicles^1^. From January to December 2023, the number of public chargers increased by 0.93 million. The number of public chargers increased by 51% year-on-year. The number of private piles increased by 2.457 million compared with the end of 2022. The number of private chargers increased by 69% year-on-year. Currently, based on the calculation of one public charger equivalent to three private chargers, the ratio of chargers to pure electric vehicles in the incremental market in China in 2023 has reached a 1:1 ratio^2^. The moderate advance development of chargers has resulted in underutilization, leading to overall losses in the operation of charging facilities.

A reasonable and comprehensive charger network is one of the key requirements for EV users. By studying the localization and capacity determination, the optimal layout of EV chargers can be determined, allowing users to conveniently find charging facilities in different areas and locations, thereby enhancing the charging experience and convenience for users. Additionally, through scientifically and reasonably positioning and determining the capacity of EV chargers, existing power resources can be fully utilized, avoiding energy waste and unnecessary overinvestment.

### Related research literature and research gaps

Through a well-planned layout, charging stations and/or chargers can better serve a wide range of users, reduce user waiting time, and improve charging efficiency^3–5^. Optimized layout ensures the maximum utilization of charging station capacity to meet the growing demand for electric vehicle charging. Reasonable placement of chargers in high-demand areas can reduce congestion at charging stations and improve the overall availability of the chargers^6–8^. Furthermore, through a reasonable layout of the chargers, it is possible to better coordinate the relationship between the power grid and charging stations, thereby improving energy utilization efficiency and supporting urban planning and sustainable development. EV is a flexible electrical load. By implementing intelligent scheduling and load management of EV chargers, it is possible to avoid energy supply instability and reduce the impact on the power system^9–16^.

The localization planning of chargers is influenced by multiple factors, including economic, policy, environmental, and social considerations. Currently, most research efforts are focused on the control strategies of chargers or the design of related systems/components^17–19^. There is relatively less research conducted on data algorithms for macro-level site selection and capacity determination. A grey decision-making model was proposed for the location of EV charging stations in^20^, the optimization equations and algorithms were clearly presented. However, the granularity of grid partitioning was not fine enough, and when the amount of data is sufficiently large and the granularity is sufficiently high, the algorithm may not be applicable. High-resolution data of EV charging stations was presented in^21^, which can be used for identifying charging behavior. A general location and sizing planning method of EV charging stations was proposed in^22^, and dynamic real-time data was utilized for optimal location planning instead of statistical data, however, the specific algorithm optimization efficiency has not been mentioned.

In recent years, some studies have also focused on the planning of charger localizations. In^23^, a modeling approach for public charging demand estimation and charging station location optimization has been proposed. The main objective of the method is to reposition existing charging stations with the goal of maximizing the overall coverage rate of charging demand. Furthermore, no specific solution algorithm was involved; it was only mentioned the use of the commercial solver Gurobi for the solution. In^24^, a method for siting EV charging stations based on improving accessibility has been proposed, the method first evaluated the target value and then optimizes the position based on available coordinates, with the objective of enhancing pedestrian accessibility. However, no specific solution algorithm or solver was mentioned. In^25^, a robust-optimization-method-based optimization framework for optimal sizing and siting of EV charging stations in power distribution systems has been presented, the uncertainties in electricity load were considered and appropriate strategies can be taken to tackle the uncertainties while keeping the system operation stable and gaining financial profit. However, the siting of EV charging stations is limited to the structure of the distribution network lines rather than geographical locations. In^26^, a data-driven approach has been proposed to sub-optimally allocate charging stations for EVs in early stages. The objective is to promote the transition of EVs from traditional cars, and the approaches are essentially the methods for optimizing, not algorithms for solving computational problems. In^27^, a data-driven approach has been proposed to solve the EV charging station location-routing problem, and the optimization problem can be formulated as a partition-based clustering problem with size constraints. The results and conclusions are also reliable. The comparison of the research works is presented in Table 1.

**Table 1:** Comparison of the research works.

Research works	Objective	Key method	Model type	Key algorithm or solver
^23^	Maximizing the overall coverage rate of charging demand	CMCLP model	Optimization	Gurobi
^24^	Maximizing the value of primary fields and enhancing pedestrian accessibility	Primary field determination and iterative analysis	Optimization	/
^25^	Maximizing the profit with optimal sizing and siting of EVCSs	Linearized Robust Approach	Optimization	/
^26^	Promoting the transition of EVs from traditional cars	δ-nearest model and K-nearest model	Optimization	GEVCSP
^27^	Developing an efficient routing strategy	Formulating the partition-based clustering problem with size constraints	Cluster	SSDF, LSDF
This paper	Minimizing the overall cost	Linearized the constraints	Optimization	GA, SOA, MILP

*CMCLP* capacitated maximal coverage location problem, *EVCSs* electrical vehicle charging stations, *GEVCSP* reedy EV charging station placement, *SSDF* smallest size difference first, *LSDF* largest size-difference first.

### Main contributions

**Figure 1:**
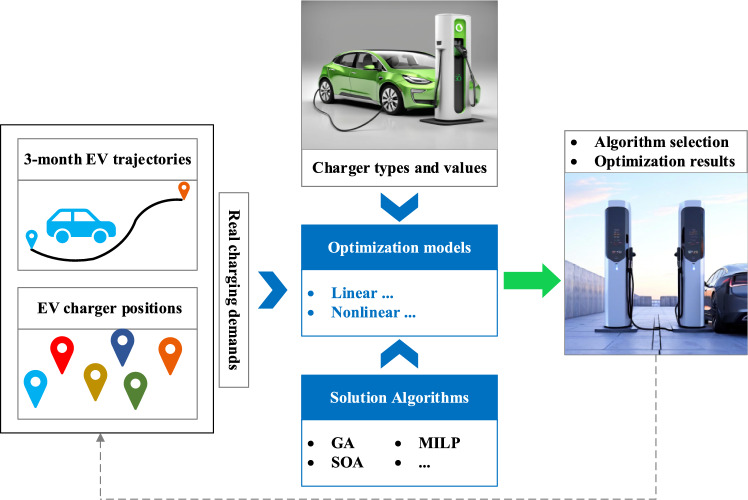
Framework and main contributions of the paper.

Due to the large scale of the localization and capacity determination problem of EV chargers, motivated by the previous researches, this paper aims to compare the performance of different algorithms at the algorithmic level under the influence of large-scale data and identify algorithms suitable for a certain data scale. Firstly, the spatiotemporal distribution data of EVs and chargers from a certain region were collected continuously for three months. Next, an optimization model was established with the objective of minimizing the total investment cost of chargers and the constraint of meeting the charging demands of all EVs. Then, the feasibility of transforming nonlinear problems into linear problems was explored, and a model was established. Finally, the performance of three common optimization algorithms, genetic algorithm (GA), surrogate optimization algorithm (SOA), and mixed integer linear programming (MILP) algorithm, respectively, has been compared in terms of result accuracy and computational time under different amount of data sets. Figure 1 present the framework and the main contributions of the paper which are listed as follows:

- (1) The city-level optimization algorithms for localization and capacity determination of EV chargers have been investigated. To the best of our knowledge, this is the first comparison at the algorithmic level of large-scale optimization for EV chargers and it identifies suitable algorithms for a certain data scale.
- (2) A three-month investigation has been conducted on city-level EV travel trajectories to provide a more effective assessment of the actual charging demand of EV users.
- (3) EV trajectories and the amount of the data have been proven to have a significant impact on the optimization results. The selection of large-scale data optimization algorithms is also related to the characteristics of the data itself. Some intelligent algorithms tend to fall into local optima when dealing with scattered data.

## Methods

The problem of localization and capacity determination of EV chargers can be treated as a typical optimization problem, in the paper, it can be described as minimizing the overall cost while satisfying the charging demands of EVs. In real-world planning, the typical goal is to install chargers of certain power types in specific areas. In the subsequent assumptions of this paper, geographical areas are divided into grids and numbered, and each charger has its own power rating, price, and number.

### Objective function

There are numerous direct and indirect factors that influence the overall cost of the location and capacity determination of EV chargers. In order to avoid excessive complexity, this paper only analyzes the initial investment costs of the EV chargers from the perspective of the power grid company. Thus, the objective function can be presented in (1), where *C*_all_ represents the overall cost, and *c*_*ij*_ represents the cost of *i*-th charger in *j*-th region.

1 $$\min \begin{array}{*{20}c} {} & {C_{{{\text{all}}}} = \sum\limits_{i} {\sum\limits_{j} {c_{ij} } } } \\ \end{array}$$

### Charging demands

Considering the geographical dispersion of EV users, each region’s chargers must ensure the charging demands of all EVs within that region. Based on the tracking records of EV driving trajectories within each region over a period of time, data such as the energy consumption per unit distance for each EV and the total charging demands can be obtained, as shown in (2, 3), where *pd*(*k*) is the energy consumption per unit distance of *k*-th EV, *P*_*con*_(*k*) is the total energy consumption during the derived distance of *k*-th EV, *d*(*k*) is the derived distance of *k*-th EV, *de*(*k*) is the charging demand of *k*-th EV, *η*(*k*) is the charging efficiency of *k*-th EV.

2 $$pd(k) = \frac{{P_{con} (k)}}{d(k)}$$

3 $$de(k) = \frac{pd(k)}{{\eta (k)}}$$

The constraints on the charging demand of the EVs involve at least two meanings. Firstly, the EVs should have sufficient remaining battery capacity to reach the nearest charger, and this is the fundamental requirement and is generally fulfilled. Secondly, the satisfaction of the charging experience for EV users, and this is a highly subjective constraint with multiple influencing factors. For simplicity, it is defined here as an average daily charging duration not exceeding 2 h. Assuming that the hypothetical cost of a single charger is *c*_sp_(*i*) and the power of a single charger is *p*(*i*), the charging demand constraints within the time period *T* (days) can be presented in (4), where *P*_charger_ represents the set of each charger power. It is to be noted that, there must be other ways to define the EV user satisfaction demands, for other definitions of the satisfaction demands, the constraints need to be modified according to specific circumstances. In all subsequent indices for the EV charger optimization in this paper, the charging demand constraint (4) needs to be satisfied.

4 $$\begin{gathered} s.t.\frac{de(k)}{{\sum {p(i)} }} \le 2T \hfill \\ { }p(i) \in P_{{{\text{charger}}}} \hfill \\ \end{gathered}$$

### Algorithms

There are two main challenges in algorithm design and selection: (1) large amount of data; (2) non-linear integer programming. Currently, there is a wide range of development and applications in the field of big data. However, when it comes to algorithms that need to operate within limited computational power and reasonable computation time, the options are quite limited.

#### Genetic algorithm

Genetic algorithm (GA) is a kind of search algorithm inspired by the theory of natural evolution. After years of development, GA has become a relatively mature computing method. GA begins by creating a random initial population^28–30^. As the localization and capacity determination problem in the paper is essentially an integer programming problem, the algorithm proposed in reference^28^ is directly employed for solving it.

#### Surrogate optimization algorithm

**Figure 2:**
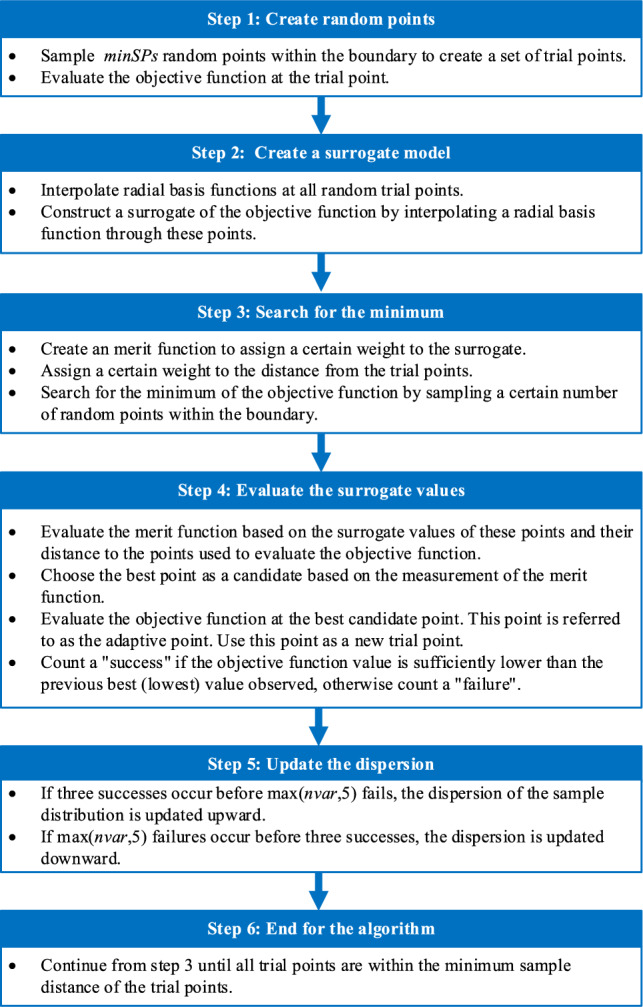
Detailed steps of SOA, where *minSPs* represents the minimum number of surrogate points, *nvar* represents the number of variables.

Surrogate optimization algorithm (SOA) is specifically designed to tackle large-scale computations^31,32^. During the execution process, SOA alternates between the “surrogate construction” and “searching for the minimum value” stages. At the surrogate construction stage, the algorithm constructs sample points from a quasi-random sequence and evaluates the objective function for these points. At the searching for the minimum value stage, the algorithm searches for the minimum value of the objective function by sampling thousands of random points within the boundaries. The evaluation of the objective function is based on the surrogate values of these points and their distances from the previously evaluated points of the objective function. When all the search points are close enough to the previously evaluated points of the objective function, the algorithm will stop the searching for the minimum value stage. The detailed steps of the algorithm are presented in Fig. 2.

#### Mixed-integer linear programming algorithm

Mixed-integer linear programming (MILP) Algorithm is a kind of algorithm designed to solve problems where the objective function is a linear equation, the constraints are linear constraints, and the optimization variables contain integer variables^33–35^. The focus of integer programming is branch-and-bound calculation^33^, which constructs a series of subproblems that attempt to converge to a solution of the MILP. The subproblems give a series of upper and lower bounds on the solution of the objective function. Firstly, MILP will still be solved as a linear programming problem without integer constraints as the root node. From the starting bounds, the branch-and-bound method constructs new subproblems by branching from the root node. Branching steps are performed heuristically according to one of several rules. Each rule is based on the idea of splitting the problem by restricting a variable to be less than or equal to an integer *J* or greater than or equal to *J* + 1.

It is to be noticed that MILP cannot be directly used for the localization and capacity determination problem of the chargers. Therefore, it is necessary to make adjustments to the original optimization problem in order to transform it into a linear optimization problem. Firstly, the objective function is modified as shown in (5), where *x* represents the number vector of the chargers, *f*_1_ represents the cost vector of the chargers.

5 $$\min \begin{array}{*{20}c} {} & {f_{1} \cdot x} \\ \end{array}$$

In this problem, since there are 7 different types of chargers with corresponding prices, the quantity of the vectors *x* and *f*_1_ both are 7 times the number of regional grids. Thus, *f*_1_ can be shown as (6).

6 $$f_{1} = \left[ {c_{{{\text{sp}}}} \left( 1 \right),c_{{{\text{sp}}}} \left( 2 \right),c_{{{\text{sp}}}} \left( 3 \right),c_{{{\text{sp}}}} \left( 4 \right),c_{{{\text{sp}}}} \left( 5 \right),c_{{{\text{sp}}}} \left( 6 \right),c_{{{\text{sp}}}} \left( 7 \right),c_{{{\text{sp}}}} \left( 1 \right),c_{{{\text{sp}}}} \left( 2 \right),c_{{{\text{sp}}}} \left( 3 \right),c_{{{\text{sp}}}} \left( 4 \right),c_{{{\text{sp}}}} \left( 5 \right),c_{{{\text{sp}}}} \left( 6 \right),c_{{{\text{sp}}}} \left( 7 \right) \ldots } \right]$$

In addition, the constraints also need to be modified into linear constraints, as shown in (7), where *A* is the coefficient matrix, and *b* is the constraint vector. *A* and *b* can be present in (8, 9).

7 $$s.t.\begin{array}{*{20}c} {} & {A \cdot x} \\ \end{array} \le b$$

8 $$A = - [{\text{eye}}(Ncc)\begin{array}{*{20}c} {} & {{\text{eye}}(Ncc)} \\ \end{array} \begin{array}{*{20}c} {} & { \ldots \begin{array}{*{20}c} {} & {{\text{eye}}(Ncc)} \\ \end{array} } \\ \end{array} ]$$

9 $$b = - \frac{de(j)}{{2T}}$$

In (8), eye(*N*_*cc*_) represents a diagonal matrix with size *N*_*cc*_ × *N*_*cc*_, *N*_*cc*_ represents the total number of the geographical regions and all the values of diagonal elements are 1. The size of *A* is *N*_*cc*_ × 7*N*_*cc*_. In (9), *de*(*j*) represents the charging demands of all EVs within *j*-th region.

## Results

**Figure 3:**
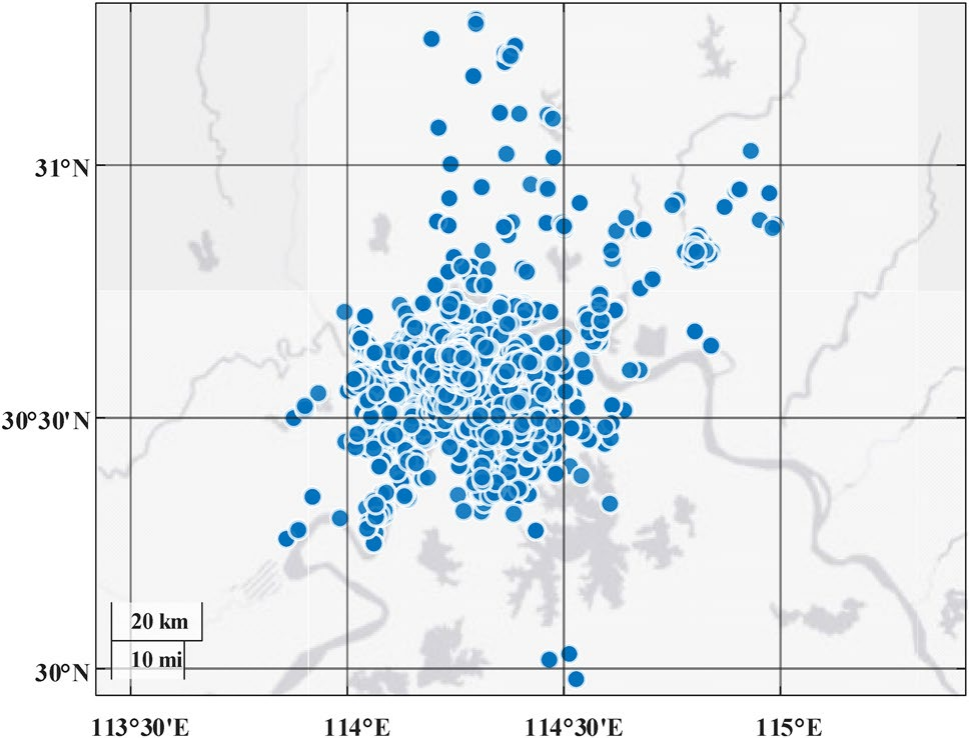
Distribution of the collected chargers. In the figure, the horizontal and vertical axes represent longitude and latitude, respectively. The blue dots indicate the geographical coordinates of the charger locations, while the dark gray color represents rivers and lakes.

In order to better serve EV users and plan the capacity, quantity, and location of the chargers more effectively, the driving trajectory data of selected EV users in a central Chinese city were collected for a period of three months. The dataset includes 33,374 EVs, 9,145,835 valid driving records, and 5,122 chargers. Due to privacy protection reasons, specific user data cannot be disclosed. Partial data that has undergone anonymization is presented in Table 2. “ID” represents the identification number of the EV, where the same number indicates multiple records. “Start time” and “Stop time” indicate the start and stop times of the vehicle, using Portable Operating System Interface (POSIX) time^36^. “Start SOC” and “Stop SOC” represent the battery state of charge (SOC) at the start and stop times of the vehicle, respectively. “Start longitude” and “Start latitude” represent the longitude and latitude at the start time of the vehicle. “Stop longitude” and “Stop latitude” represent the longitude and latitude at the stop time of the vehicle. The distribution of the collected chargers can be used as the initial state, as shown in Fig. 3.

**Table 2:** Partial data of the driving trajectory of selected EV users.

ID	Vehicle use	Start time	Stop time	Start SOC	Stop SOC	Start longitude	Start latitude	Stop longitude	Stop latitude
1	4	1.61E+12	1.61E+12	64	61	114.xxxx	30.xxxx	114.xxxx	30.xxxx
1	4	1.61E+12	1.61E+12	60	59	114.xxxx	30.xxxx	114.xxxx	30.xxxx
1	4	1.61E+12	1.61E+12	59	56	114.xxxx	30.xxxx	114.xxxx	30.xxxx
…	…	…	…	…	…	…	…	…	…

In order to simplify the calculation, the geographical region under study was divided into grids, the total number of grids is *N*_*cc*_ in the case, *N*_*cc*_ = 33489, and the latitude and longitude coordinates were evenly divided into 183 parts. After statistical analysis of these chargers, all chargers were divided into 7 categories. The power of a single charger is 7, 60, 80, 120, 160, 180, and 300 kW respectively, and the price is 3000, 25000, 32000, 39000, 52000, 59000, and 72000 yuan (RMB) respectively. All the simulation results in this paper were obtained running on a computer with an AMD Ryzen Threadripper 3970X 32-Core Processor (CPU) and 64 gigabytes (GB) of Random-access memory (RAM). An additional 1.9 terabytes (TB) of virtual memory were also allocated for the computations.

### Results of genetic algorithm

**Figure 4:**
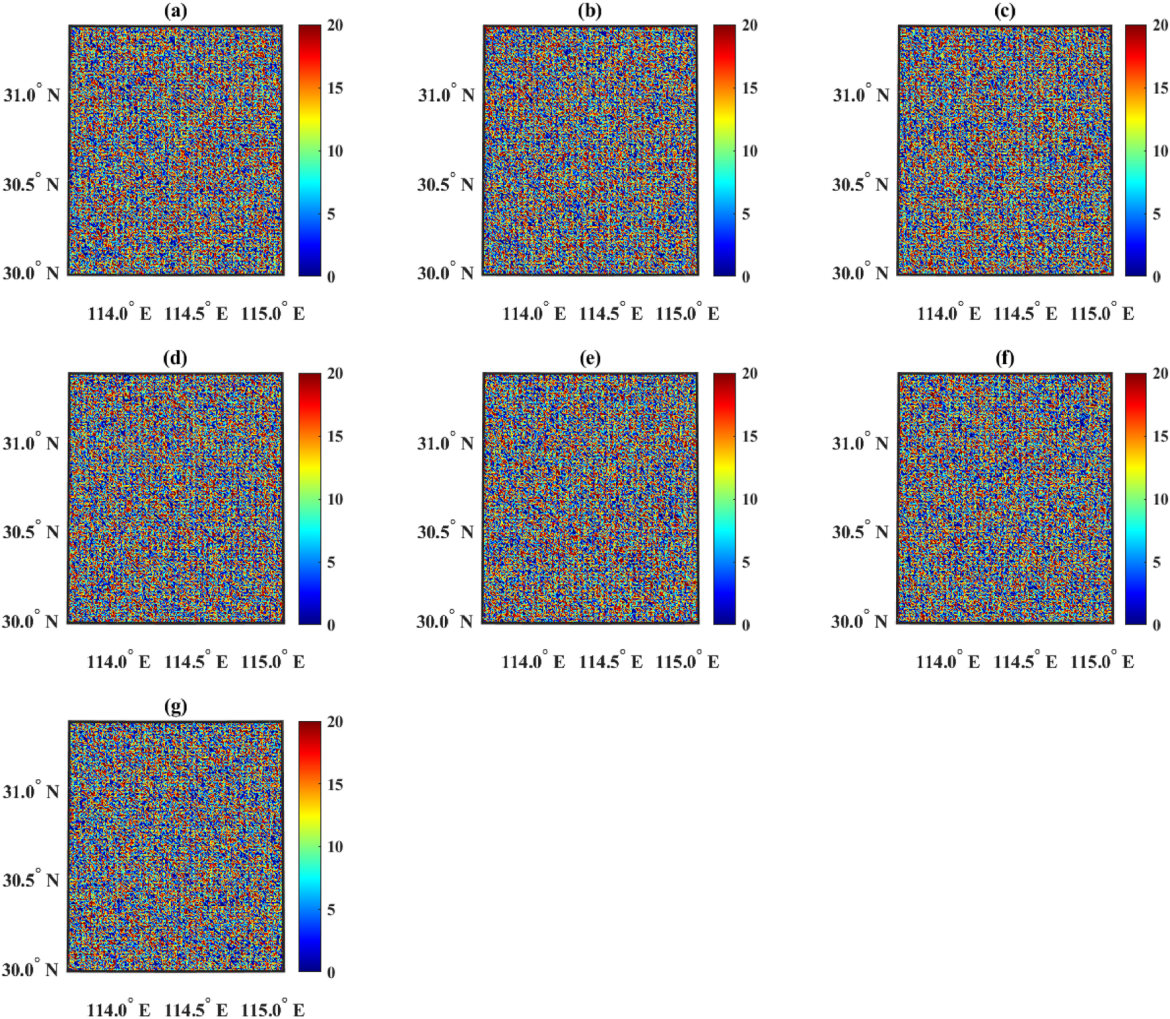
Distribution of chargers after GA optimization. Figures (**a**–**g**) show the distribution of chargers of categories 1 to 7 respectively, and the horizontal and vertical axes represent longitude and latitude, respectively. The color scale indicates the number of chargers.

In the simulation of the Genetic algorithm (GA), parallel computing with 32 cores was utilized. After a duration of 13623.73 s, the results were obtained, as shown in Fig. 4. It is obvious that the distributions shown in Fig. 4 are too scattered. The reason is that the algorithm fell into a local optimal solution and did not obtain the correct optimal solution globally. Overall, the solution time of GA was too long and the correct solution was not obtained.

GA is an optimization algorithm inspired by the principles of natural selection and genetics. It works by simulating the process of evolution, including selection, crossover, and mutation, to iteratively improve potential solutions to a problem. GA can perform global search in the search space through random selection, crossover, and mutation operations. It can automatically adjust the search direction, thus progressing faster towards the optimal solution. However, when the search space of the problem is extremely large, GA may be limited by the search space, unable to find better solutions, and thus may get trapped in local optima. Specifically, in the problem of localization and capacity determination of EV chargers, the resulting location may be as scattered as shown in Fig. 4.

### Results of surrogate optimization algorithm

**Figure 5:**
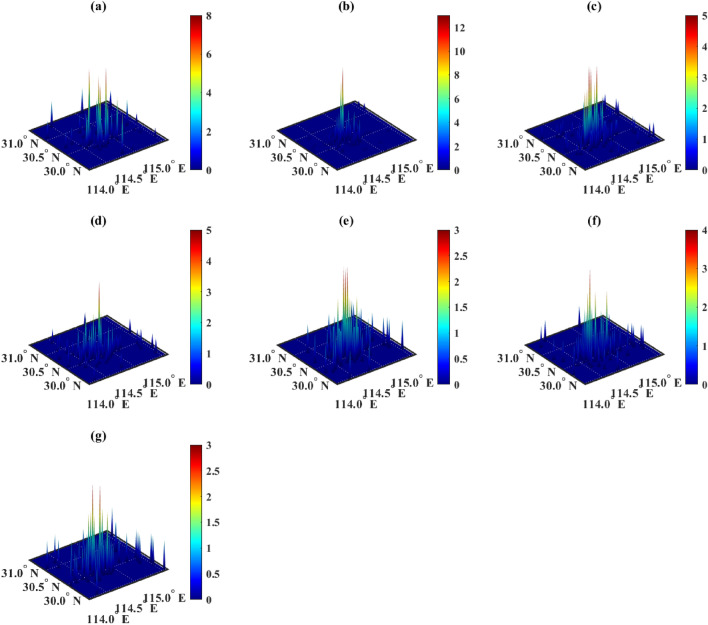
Distribution of chargers after SOA optimization. Figures (**a**–**g**) show the distribution of chargers of categories 1 to 7 respectively, and the horizontal and vertical axes represent longitude and latitude, respectively. The color scale indicates the number of chargers.

The algorithmic environment for surrogate optimization algorithm (SOA) is the same as that for GA. In the case of complete data amount, SOA did not produce any results and reported an “out of memory” error. Then, 10% of the original data were randomly selected and the calculation was re-executed, and the results obtained are shown in Fig. 5. The results in Fig. 5 are noticeably better than those in Fig. 4. The regions with a higher demand for chargers are concentrated in the central urban area. And the final calculated minimum cost was 3.01 million RMB.

### Results of mixed-integer linear programming algorithm

**Figure 6:**
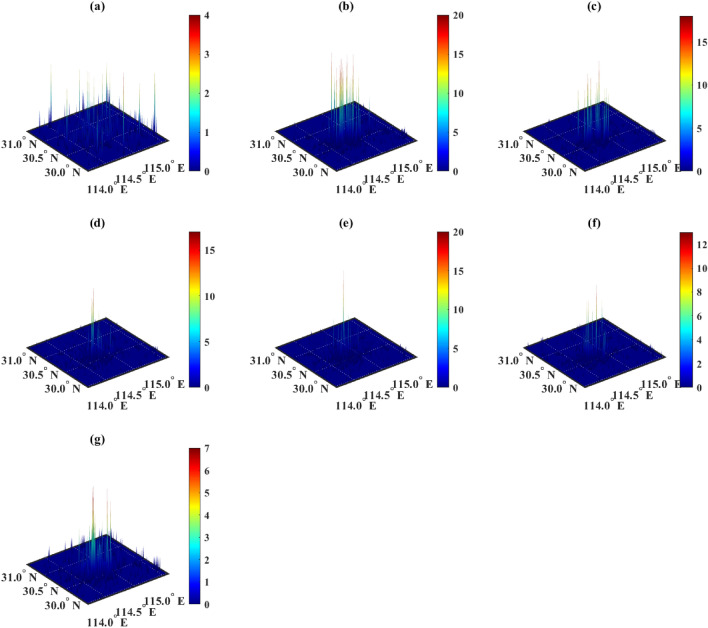
Distribution of chargers after MILP optimization. Figures (**a**–**g**) show the distribution of chargers of categories 1 to 7 respectively, and the horizontal and vertical axes represent longitude and latitude, respectively. The color scale indicates the number of chargers.

The algorithmic environment for MILP is also the same as that for GA. The difference is that MILP performs an improved version of linear programming. After a duration of 14182.57 s, the results were obtained, as shown in Fig. 5. The results in Fig. 6 are similar to Fig. 5. The regions with a higher demand for chargers are concentrated in the central urban area. And the final calculated minimum cost was 34.62 million yuan.

#### Influence of the geographical location of EVs

Furthermore, an additional perturbation factor *G*_*p*_ related to geographical location has been introduced. Firstly, the initial distribution of EVs was encoded based on the geographical grid regions, with the minimum value of the encoding being 1 and the maximum value being *N*_*cc*_. Overlaying the *G*_*p*_ value onto the grid number where the EV is located is equivalent to perturbing the position of the EV. The larger the *G*_*p*_ value, the greater the perturbation, and the more dispersed the position of the EV will be.

Firstly, the *G*_*p*_ value was set as a random integer between ± 5 and added to the encoding of the initial distribution of EVs. After a computation time of 13785.51 s, the distribution of chargers results in a similar pattern as depicted in Fig. 5. This time, the minimum cost of the chargers was 49.19 million yuan. Thus, additional different *G*_*p*_ values have been used for further computational analysis, the results are presented in Table 3. It can be observed that as the *G*_*p*_ value increases, there is no significant pattern of change in computation time. However, the minimum cost of the chargers tends to increase. The reason is that the increase in *G*_*p*_ value directly leads to more dispersed EV locations, which will result in the need for more low-power chargers and fewer high-power chargers, thus weakening the price advantage of high-power and cost-effective chargers.

**Table 3:** Comparison of computation time and minimum cost of EVs under disturbances in different geographical locations.

*G*_*p*_	Computation time (seconds)	Minimum cost (million yuan)
± 5	13785.51	49.19
± 10	11949.58	56.38
± 50	13387.20	90.66
± 100	12677.02	109.04
± 200	12388.49	103.93
± 400	12379.20	114.08

#### Influence of the amount of EVs

**Figure 7:**
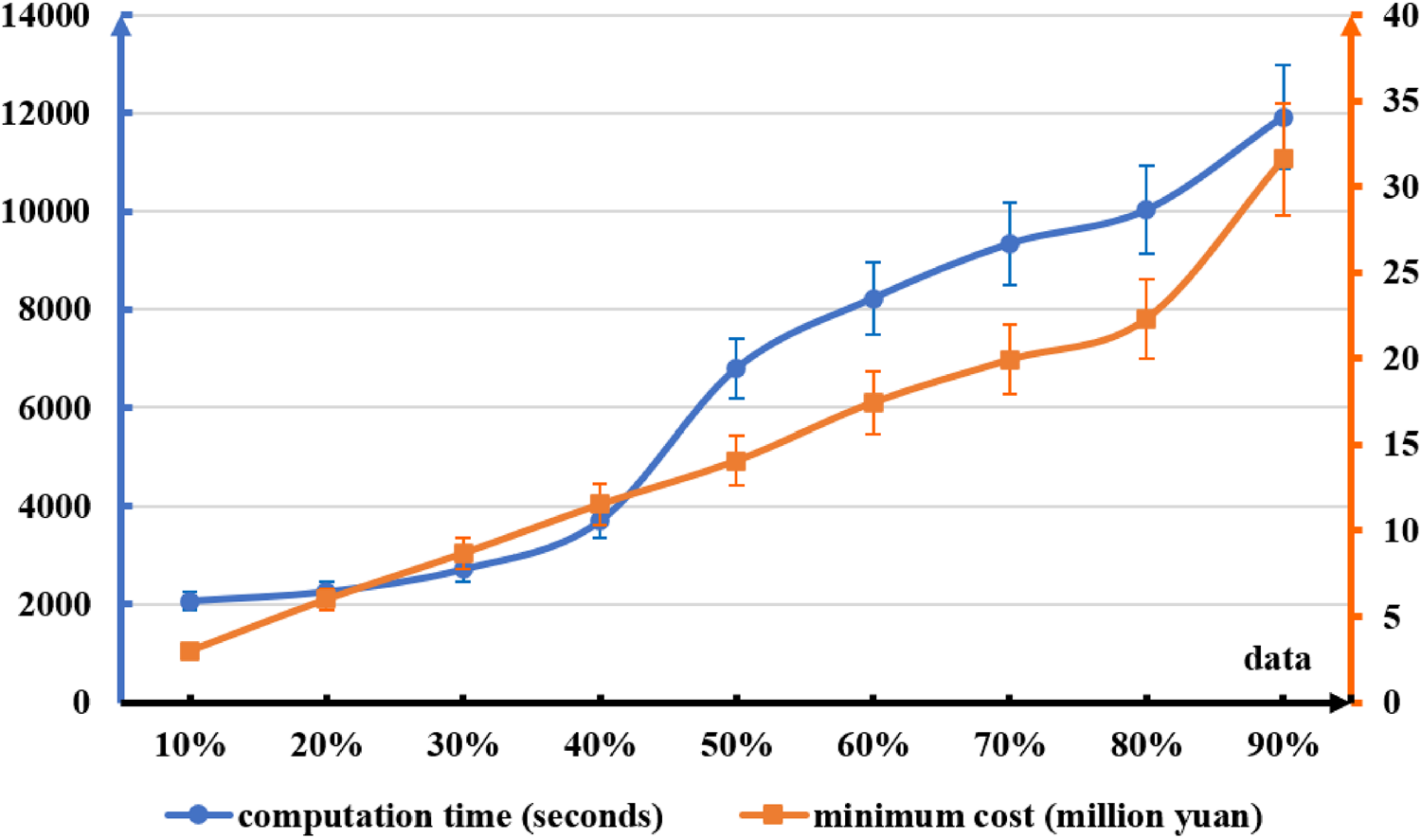
Comparison of computation time and minimum cost of EVs under different amount of EVs.

The amount of EVs is the most significant factor. For further analysis, 10% to 90% of the total amount of data was randomly selected, and MILP was reused for calculation. The results are shown in Fig. 7. As the amount of EVs increases, both the computation time and the minimum cost increase. Within the range of 10 to 80% of the data amount, the minimum cost grows almost linearly with the data amount, and the growth rate increases significantly when the data amount reaches 80 to 90%. In the range of 10 to 40% of the data amount, the computation time increases slowly with the data amount; above 40% of the data amount, the computation time increases relatively quickly with the data amount.

### Model validation

Firstly, the data used in the paper comes from real data collected over a period of 3 months, which ensures the authenticity and reliability of the data source. Furthermore, MILP calculations were performed with 10% of the data amount, and the calculation results (including the minimum cost and the distribution of chargers) were consistent with SOA under the same calculation conditions. In this way, the accuracy of the results was cross-validated by SOA and MILP. In addition, from the comparison of different *G*_*p*_ values, it can be seen that the more disorderly the distribution of EVs, the higher the minimum cost; from the comparison of computation time and minimum cost under different data volumes, it can be seen that as the data amount increases, the computation time and minimum cost both increase accordingly, and these results are generally in line with expectations.

## Discussions and future works

From the comparison of the results obtained by three different algorithms, it can be seen that in the case of large-scale simulation optimization, GA is prone to falling into local optima, SOA consumes excessive memory. In comparison, simplifying complex nonlinear optimization problems into linear problems as much as possible is a feasible approach. From the results, it can be observed that MILP performs the best among the three algorithms.

Specifically, GA has the highest efficiency and possesses a relatively mature algorithmic framework. It is highly applicable and capable of handling problems involving linear, nonlinear, integer, and non-integer variables. However, when dealing with large-scale nonlinear integer optimization problems, it is prone to falling into local optima and producing incorrect results. SOA has similarly excellent applicability to GA and is even more likely to produce correct results. However, it suffers from high memory consumption. The algorithm was originally designed to handle large-scale computations more effectively, but due to its memory usage issues, the practical application of SOA may be severely restricted. The applicability of MILP is not as extensive as GA and SOA. However, if the nonlinear problem can be formulated as a linear problem, the results obtained from MILP will be more reliable.

**Figure 8:**
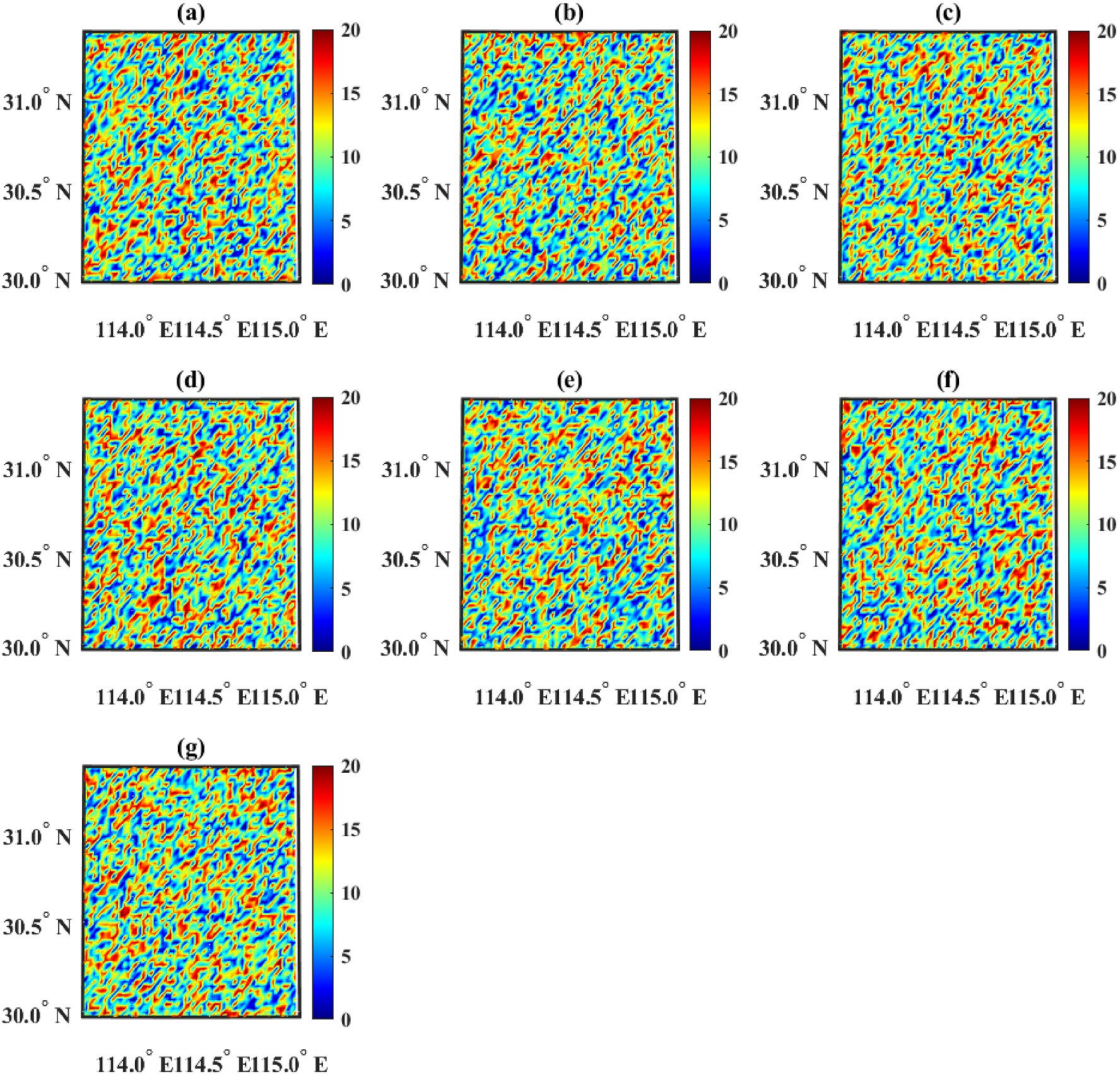
Distribution of chargers after GA optimization on the linearized problem. Figures (**a**–**g**) show the distribution of chargers of categories 1 to 7 respectively, and the horizontal and vertical axes represent longitude and latitude, respectively. The color scale indicates the number of chargers.

In order to further test the algorithm performance of GA, simulations are conducted using GA on the linearized problem. Firstly, in the case of complete data, GA reports an “out of memory” error. Then, the data amount was reduced to 10% of the original data, and after running for 417.5 s, the results are obtained as shown in Fig. 8. GA once again falls into a local optimum. The reason why GA is prone to falling into local optima is related to its iterative algorithm mechanism.

The paper compared the performance of different algorithms in solving the large-scale optimization problem of EV charger localization and capacity determination. As a common and typical intelligent algorithm, GA simulates the genetic and evolutionary processes in nature. By continuously iterating, it can gradually search for optimal solution spaces and has a certain level of efficiency and robustness in solving complex optimization problems. In contrast, MILP is not as “intelligent”. It is generally believed that for large-scale, complex problems, MILP algorithms may face challenges of high computational complexity. The level of intelligence in SOA lies between the two. The results indicate that when dealing with optimization problems with scattered data, such as the localization and capacity determination of EV chargers, overly “intelligent” algorithms may not necessarily yield the best results. The limitation of the paper lies in its inability to exhaust all intelligent algorithms. With further research into data characteristics and the rapid development of artificial intelligence, there will certainly be intelligent algorithms in the future that are more effective and efficient than GA and MILP. Based on a thorough investigation of data structures, the authors will focus on the development of adaptive algorithms and self-organizing algorithms in the future. This will enable algorithms to automatically adjust their parameters and structures according to changes in the environment and tasks.

## Conclusions

Based on considering the spatiotemporal distribution of EVs, this paper conducted research on the optimization problem of chargers at the city level. In the planning of city-level EV chargers, MILP took 14182.57 s to calculate the minimum cost of 34.62 million yuan. After retaining only 10% of the original data amount, SOA took 87651.34 s to calculate the minimum cost of 3.01 million yuan. The concrete conclusions can be drawn:

- (1) The city-level charger optimization problem can be solved through SOA or through MILP after converting the problem into a linear one.
- (2) GA is prone to falling into local optima and is not suitable for the optimization of localization and capacity determination of chargers in larger scales.
- (3) SOA requires significant memory consumption, so the issue of memory usage needs to be carefully considered when using it directly.
- (4) The deviation of EV trajectories and the amount of the data have a significant impact on the optimization results of MILP. The more dispersed the spatiotemporal distribution of EVs, the higher the overall minimum cost of the chargers. As the amount of EVs increases, both the computation time and the minimum cost increase.

## Data Availability

The datasets generated and/or analysed during the current study are not publicly available due to sensitive information protection, but are available from the corresponding author on reasonable request.

## Code availability

The code used in this study is not publicly available due to confidentiality agreements with State Grid. However, detailed descriptions of the algorithms and methods used are provided in the Methods section of the manuscript. Researchers interested in the code for collaborative purposes can contact the corresponding author.
